# Exploring the Role of Self-Control Across Distinct Patterns of Cyber-Deviance in Emerging Adolescence

**DOI:** 10.1177/0306624X231220011

**Published:** 2024-01-04

**Authors:** Tyson Whitten, Jesse Cale, Russell Brewer, Katie Logos, Thomas J. Holt, Andrew Goldsmith

**Affiliations:** 1 Center for Law and Justice, Charles Sturt University, Port Macquarie, NSW, Australia; 2 Discipline of Psychiatry and Mental Health, University of New South Wales, Sydney, NSW, Australia; 3 School of Criminology and Criminal Justice, Griffith University, Brisbane, QLD, Australia; 4 School of Social Sciences, University of Adelaide, Adelaide, SA, Australia; 5 School of Criminal Justice, Michigan State University, Michigan, USA; 6 Centre for Crime Policy and Research, Flinders University, Adelaide, SA, Adelaide

**Keywords:** self-control, adolescent cyber-deviance, latent class analysis, general theory of crime

## Abstract

A disproportionally large number of adolescents engage in cyber-deviance. However, it is unclear if distinct patterns of adolescent cyber-deviance are evident, and if so, whether and to what extent low self-control is associated with different patterns of cyber-deviance. The current study addressed this research gap by examining the relationship between self-control and distinct latent classes of adolescent cyber-deviance net of potential confounders among a cross-sectional sample of 1793 South Australian adolescents. Four latent classes were identified, each characterized by varying probabilities of involvement in six types of cyber-deviance that were measured. The *versatile* class (*n* = 413) had the lowest average level of self-control, followed by the *harmful content users* (*n* = 439) and *digital piracy* (*n* = 356) classes, with the *abstainer* class (*n* = 585) characterized by the highest self-control. Analysis of covariance indicated that the *abstainer* group had significantly higher self-control than other classes of cyber-deviance. Although the *versatile* class had noticeably lower average self-control scores than the *harmful content users* and *digital piracy* groups, this difference was not significant after correcting for multiple comparisons. Collectively, these findings suggest that self-control appears to distinguish between those who do and do not engage in cyber-deviance but may not distinguish between distinct patterns of cyber-deviance net of other factors.

## Introduction

Young people aged 12 to 17 years are disproportionally more likely to be involved in some form of cyber-deviance relative to other age groups (Aaltonen & Salmi, 2013; Kim et al., 2017; McGuire & Dowling, 2013). Among this group of adolescents, males are more likely than females to engage in most forms of cyber-deviance (Donner, 2016; Shapka et al., 2018; Sorrentino et al., 2019), although some evidence suggests that cyberbullying and online harassment may be more common among adolescent females (Balakrishnan, 2015; Barlett & Coyne, 2014; Faucher et al., 2015). Furthermore, race and socioeconomic disadvantage also appear to be associated with hacking, cyberbullying, and digital piracy (Gunter et al., 2010; Low & Espelage, 2013; Marcum, Higgins, Ricketts, & Wolfe, 2014). Certain patterns of technology use are also linked to adolescent engagement in cyber-deviance, including frequency of digital device and internet use (Aaltonen & Salmi, 2013; Bae, 2017; Holt, Cale, et al., 2021) and lack of parental monitoring while online (Ang, 2015; Kowalski et al., 2014). Importantly, a growing body of research suggests that low self-control also plays a key role in cyber-deviance much the same way it does with offline antisocial behavior among adolescents (Holt et al., 2012; Holt, Cale, et al., 2021; Holt, Holt, et al., 2021).

The widespread use of digital technologies has created new opportunities for deviance that range from extensions of traditionally offline antisocial behaviors conducted on a digital platform (i.e., cyber-enabled crimes such as fraud, theft, bullying, and sexual harassment), to new acts that specifically exploit vulnerable computer systems and infrastructure (i.e., cyber-dependent crimes such as hacking, distributed denial of service attacks, and the dissemination of malicious software) (McGuire & Dowling, 2013). Research on adolescents has typically examined engagement in online antisocial behavior either by examining a specific form of cyber-deviance, most often cyberbullying (e.g., Li et al., 2016; Virgara & Whitten, 2022), or combining multiple forms of online antisocial behavior into a single indicator to reflect general cyber-deviance (e.g., Bae, 2017). To date, no study has examined if distinct categories of online antisocial behaviors cluster together to form unique patterns of cyber-deviance, and whether and how low self-control may be associated with different patterns of cyber-deviance perpetration beyond other explanatory factors.

### Low Self-Control, Offline Deviance, and Cyber-Deviance

According to Gottfredson and Hirschi (1990), low self-control is the key trait underpinning all acts of antisocial behavior given the presence of opportunities to do so. They argued that self-control is developed from mid-to-late childhood primarily via parental socialization and remains relatively stable throughout the life-course. Although the stability of low self-control is a key point of debate in the literature (e.g., see Burt et al., 2014; Hay & Forrest, 2006), it is generally accepted that individuals characterized by low self-control tend to be more impulsive in their actions, seek instant gratification, and engage in more risk-taking behaviors, particularly during adolescence (e.g., Casey & Caudle, 2013; Shulman et al., 2016; Steinberg & Chein, 2015).

Several meta-analyses provide strong evidence across adolescent and adult samples of the robust relationship between low self-control and engagement in various offline antisocial behaviors (de Ridder et al., 2012; Pratt & Cullen, 2000; Vazsonyi et al., 2012). For example, Vazsonyi et al. (2017) found that across 99 studies which examined diverse adolescent/adult cross-cultural populations, low self-control was related to physical violence, substance use, organizational dishonesty, theft, and general crime and deviance (i.e., engagement across various behaviors), with the strongest associations uncovered among younger populations. Similar findings were also reported in a more recent meta-analysis of 255 effect sizes across 72 studies demonstrating that low self-control moderately predicted antisocial behaviors and mediated the effect of parenting practices (Tehrani & Yamini, 2020).

In terms of cyber-deviance, low self-control appears to be associated with digital piracy (Holt et al., 2012), cyberbullying (Choi & Kruis, 2020; Li et al., 2016; Vazsonyi et al., 2012), and hacking (Back et al., 2018; Holt et al., 2020; Holt, Cale, et al., 2021; Marcum, Higgins, Ricketts, & Wolfe, 2014). Studies have also found links between low self-control and cyber risk-taking behaviors, such as sexting and viewing pornography (Holt et al., 2012; Holt, Holt, et al., 2021; Marcum, Higgins, & Ricketts, 2014; Wachs et al., 2017). Finally, several studies have found evidence of a relationship between low self-control and general cyber-deviance, consisting of a combined indicator comprising most of the latter categories and, in some cases, additional ones such as online fraud and deception (Bae, 2017; Donner et al., 2015; Rodríguez-de-Dios et al., 2018). Although the link between low self-control and individual types of cyber-deviance are well established, no study has yet examined the relative effect of self-control among young people involved in distinct patterns of engagement across multiple types of cyber deviance.

Across studies of adolescents, low self-control is consistently positively associated with cyber-deviance even when controlling for other theoretically relevant factors such as social bonds (Back et al., 2018), perceived life stressors (Bae, 2017), and delinquent peer associations (Holt et al., 2012; Li et al., 2016; Marcum, Higgins, & Ricketts, 2014; Marcum, Higgins, Ricketts, & Wolfe, 2014). Furthermore, opportunities for online delinquency—such as frequent digital device use and lack of parental monitoring—predict cyber-deviance independent of low self-control (Back et al., 2018; Bae, 2017; Kim & Kim, 2015), and may mediate the association between low self-control and general cyber-deviance (Baek et al., 2016; Holt, Cale, et al., 2021; Holt, Holt, et al., 2021). Offline antisocial behavior may also influence the association between low self-control and cyber-deviance (Vazsonyi et al., 2012). Studies have demonstrated strong associations between offline and online delinquency, and some researchers have argued that cyber-deviance represents an extension of offline antisocial behavior (Kim et al., 2017; Seigfried-Spellar et al., 2017).

There is considerable overlap between offline antisocial behavior and cyber-deviance, particularly among adolescents (Kim et al., 2017; Seigfried-Spellar et al., 2017). Several studies have demonstrated that adolescent and young offline adult antisocial behavior is associated with various forms of cyber-deviance, including piracy, hacking, cyber-terrorism, image-based sexual abuse, and cyberbullying (Donner et al., 2015; Hinduja & Patchin, 2008). Raskauskas and Stoltz (2007) found that among a sample of 84 adolescents, perpetration of physical bullying was a strong predictor of cyberbullying, independent of age and gender. Similar findings have also been reported by Rokven et al. (2018), who found that among 320 adolescents who engaged in some form of cyber-deviance (including online harassment, fraud, and hacking), 73% admitted to also participating in antisocial behavior offline, while around half of those who engaged in antisocial behavior offline also engaged in cyber-deviance. In a large European adolescent sample, Vazsonyi et al. (2012) examined offline antisocial behavior as a facilitator of the relationship between low self-control and cyber-deviance and found that low self-control had a significant direct effect on an increased frequency of cyberbullying perpetration, but that there was also an indirect effect through frequency of offline bullying perpetration.

### Patterns of Cyber-Deviance Perpetration

There is substantial heterogeneity in patterns of engagement in offline antisocial behavior (Nieuwbeerta et al., 2011). Evidence suggests that clusters of delinquents who specialize in certain antisocial behaviors may be qualitatively different from those who specialize in other types of behaviors or are more versatile in their delinquency (Brown, 2019; McGloin et al., 2007; Spaan et al., 2020). Latent class analysis has been used to quantify these underlying groups to discern the factors that differentiate unique patterns of engagement across multiple types of antisocial behavior. This information is vital for identifying groups of delinquents who may be more receptive to certain interventions over others. However, such research has not yet been applied to cyber-deviancy. Instead, the research to date has used latent class analysis to examine if low self-control is associated with distinct classes reflecting variations in the frequency of engagement in a *single* type of cyber-deviance, typically cyberbullying (e.g., Cho & Glassner, 2021; Tian et al., 2023), and not on perpetration patterns across *multiple* types of cyber-deviance. This is an important research gap to address because adolescents tend to engage in multiple types of cyber-deviance, and therefore the relative role of self-control may differ depending on the types and patterns of perpetration (Brewer et al., 2020).

Understanding the differential association between self-control and patterns of engagement across *multiple* types of cyber-deviance may provide insights as to which patterns of online antisocial behavior may be receptive to self-control improvement programs. For example, early school-based programs that provide young people with strategies to regulate their behavior and consider the consequences of their actions are associated with a 32% improvement in self-control and 27% reduction in general offline delinquency (Piquero et al., 2016). Similar interventions also produce up to a 15% reduction in cyberbullying perpetration (Gaffney et al., 2019), although evidence regarding the impact of such programs on other types of cyber-deviance is lacking (Virgara & Whitten, 2022). If, like offline delinquency, all forms and patterns of cyber-deviance are in some way underpinned by low self-control, then existing school-based interventions may be suitable for reducing adolescent cyber-deviance, regardless of perpetration patterns.

### The Current Study

Low self-control may underly adolescent engagement in specific and general cyber-deviance. However, this association may be explained by involvement in offline antisocial behavior or the presence of opportunities for online delinquency. It is unclear whether adolescents become involved in distinct patterns of engagement in multiple types of cyber-deviance, and if so, how low self-control may be associated with these unique patterns beyond other explanations for cyber-deviance, such as engagement in antisocial behavior offline, opportunities for online misconduct (i.e., frequency of digital device engagement and time spent unsupervised online), and demographic characteristics (i.e., gender, race, and socioeconomic disadvantage). Therefore, the current study explores whether distinct patterns of cyber-deviance are evident in a sample of Australian high school students, and whether and to what extent low self-control differentiates these distinct patterns independent of other explanatory factors.

## Methodology

### Sample and Procedures

Data were drawn from the first wave of the South Australian Digital Youth Survey (DYS), a longitudinal survey of a cohort of South Australian students commencing grade 8 (the first year of high school) in 2018. The first wave of the DYS includes data on 1,921 participants (25.5% of all grade 8 enrolments; Department for Education and Child Development, 2018) from 18 government schools located within the metropolitan region (i.e., located within 100 km of the Central Business District) of a large Australian city. Surveys were paper-based and administered in class by the research team. Ethics approval was obtained through the host university Human Research Ethics Committee, with additional approval granted by the Department of Education, school principals, and classroom teachers. Parent and student consent was obtained for all participants using an opt-out procedure. Participants were informed they could withdraw from the study at any time without prejudice, and that they would be assigned a randomly generated identification code to ensure their anonymity.

### Measures

#### Cyber-deviance

Measures of cyber-deviance were adapted from previous instruments (e.g., Brewer et al., 2018; Holt et al., 2012; Holt & Kilger, 2012; Li et al., 2016) and validated for the present research. Participants were asked if, in the last 12 months, they had “never,” “less than weekly,” “about once a week,” “several times a week,” “about once a day,” or “several times a day,” engaged in a series of online deviant behaviors using either a desktop computer, laptop, tablet, or smartphone. This comprised of 29 items across six scales measuring distinct types of cyber-deviance. A variable was created for each scale indicating if participants had ever engaged in the respective online behavior within the last 12 months. These scales were:

(1) Fraud and illicit transactions (six items, α = .73): “lied about your identity,” “bought anything that might be against the law,” “sold anything that might be against the law,” “tricked another person into sending you their personal information,” “tricked another person into giving you money,” and “tricked a business or organization into sending you money, goods, or services”;
(2) Sexual activity (five items, α = .73): “seen sexual content of someone you know,” “seen sexual content of someone you don’t know,” “seen pornography on a website,” “shared sexual content of yourself,” and “shared sexual content of someone else without their consent”;
(3) Advocating violence (five items, α = .80): “seen content involving serious violence against someone you know,” “seen content involving serious violence against someone you don’t know,” “seen content involving serious violence against a group of people,” “shared content involving serious violence against another person,” and “shared content involving serious violence against a group of people”;
(4) Discrimination and bigotry (five items, α = .83): “seen content making fun of someone you know because they were different,” “seen content making fun of someone you don’t know because they were different,” “seen content making fun of a group of people because they were different,” “shared content making fun of a particular person because they were different,” and “shared content making fun of a group of people because they were different”;
(5) Intellectual property (IP) infringement (four items, α = .84): “listened to music that you think you should have paid for,” “watched a video that you think you should have paid for,” “downloaded software, games, or eBooks that you think you should have paid for,” and “shared music, videos, software, games, or eBooks with others that you think they should have paid for”; and
(6) Unauthorized access (four items, α = .84): how often participants had accessed another person’s (i) social media account and (ii) digital device, without their permission to “look at information, photos, videos, or other files” and “delete or change information or other files.”

#### Self-control

Six items adapted from the propensity for risk-taking scale from the *National Longitudinal Survey of Youth, Children and Young Adults* were used to measure self-control—with similar abbreviated measures used in other research to assess dimensions of self-control (e.g., Holt & Steinmetz, 2021). A four-point Likert scale (strongly disagree, disagree, agree, strongly agree) was used to indicate agreement with the following six statements: (1) “planning takes all the fun out of things”; (2) “I enjoy taking risks”; (3) “I often get into trouble because I do things without thinking”; (4) “I enjoy new and exciting experiences, even if it is a little frightening”; (5) “life with no danger in it would be too dull for me”; and (6) “I have to use a lot of self-control to keep out of trouble.” Scores were averaged across the six items (range 1–4; α = .72), with higher scores signifying lower self-control.

#### Opportunity

Two discrete measures were used to gauge opportunities for cyber-deviance. The first, designated *digital device use*, was derived from a 16-item scale asking participants to indicate how often (never, less than weekly, about once a week, several times a week, about once a day, several times a day) they engaged in the following online activities: (1) “searched for information using search engines”; (2) “browsed social media”; (3) “listened to music”; (4) “looked at photos or images (outside of your social media feeds)”; (5) “sent instant messages”; (6) “sent/received emails”; (7) “watched videos or movies (outside of your social media feeds)”; (8) “coding or writing software”; (9) “used software to cover your tracks online”; (10) “browsed or posted to an online forum”; (11) “shared your photos”; (12) “used the camera on any of your devices to take photos or record videos”; (13) “online banking to send or receive money”; (14) “worked on your own website or created your own content to post online (outside of social media)”; (15) “shared your videos”; and (16) “used file sharing or cloud syncing software.”

Principal components analysis was conducted to assess construct validity and reduce these 16 items into a discernible factor reflecting the extent of one’s online engagements. Four components had an eigenvalue greater than 1, and collectively explained 55.22% of the variance. The first component was retained for further inspection, as it had the highest eigenvalue (4.37), explained the most variance (27.29%), and demonstrated a clear break in the scree plot and parallel analysis. The first component had an un-rotated loading of 12 items, and a rotated loading of 8 items with coefficients greater than .3. These items were: (1) “shared your photos”; (2) “shared your videos”; (3) “used the camera on any of your devices to take photos or record videos”; (4) “browsed social media”; (5) “sent instant messages”; (6) “listened to music”; (7) “browsed or posted to an online forum”; and (8) “looked at photos or images (outside of your social media feeds).” These eight items had a Cronbach’s alpha score of .84 and a mean inter-item correlation of .39, which is optimal for scales with less than 10 items (Briggs & Cheek, 1986). Scores were averaged across the eight items, with higher scores indicating greater variety and frequency of online engagement (range 0–5).

The second measure of opportunity for cyber-deviance was *unsupervised internet use*. This comprised of a single item asking participants how often (never, rarely, sometimes, most of the time, all the time) in the last 12 months they were physically alone when using the internet. Higher scores reflect greater amounts of time spent physically unsupervised online.

#### Offline antisocial behavior

A five-item scale (α = .80) adapted from the International Self-Report Delinquency Study (Enzmann et al., 2010) measured how often (never, less than weekly, about once a week, several times a week, about once a day, or several times a day) in the last 12 months participants engaged in the following behaviors: (1) “destroyed, damaged, or vandalized property”; (2) “stolen something that didn’t belong to you”; (3) “drank alcohol”; (4) “used illegal drugs”; or (5) “beat someone up.” A binary variable was created indicating if participants had engaged in at least one type of offline antisocial behavior within the last 12 months.

#### Demographic characteristics

Participants reported their sex (male/female) and race (Caucasian/not Caucasian). Residential socioeconomic disadvantage was computed from the Socio-Economic Indexes for Areas (SEIFA) derived from the participant’s primary residential postcode. The SEIFA indexes the average income and employment status for each residential postcode in Australia, with lower scores indicating greater socio-economic disadvantage (Pink, 2013). SEIFA quintiles were produced and range from most disadvantaged (quintile 1) to most advantaged (quintile 5). This was dichotomized so that quintile 1 corresponded to most socioeconomic disadvantage as the category of interest, and quintiles 2 to 5 were the reference group.

### Analytic Strategy

Distinct patterns of cyber-deviance engagement were identified using Latent Class Analysis (LCA); a statistical method used to identify discrete classes of individuals that reflect their underlying traits based on their responses to a series of categorical variables (Lanza et al., 2007). Individuals are clustered into latent classes based on the posterior probabilities of class membership. Each latent class also encompasses a conditional probability indicating the likelihood that individuals from that class exhibit the relevant form of cyber-deviance. The latent class model makes no assumptions about the distribution of categorical indicators other than that they are independent within each latent class (otherwise known as local independence).

The LCA was conducted using the PROC LCA procedure for SAS (v. 9.4). Cases with missing data for the grouping variables were removed from the analysis (Lanza et al., 2015). A two, three, four, and five latent class solution were considered. Models were assessed using the likelihood-ratio G2 statistic, as well as the Akaike’s information criterion (AIC) and the Bayesian information criterion (BIC), both of which are penalized log-likelihood model information statistics. These statistics are used to compare model fit among competing models using the same data, with smaller values relative to the reduction in the degrees of freedom indicating better model fit and parsimony (Lanza et al., 2007).

Of the 1921 participants included in the DYS, 128 (6.7%) had missing data at random for the cyber-deviance scales (Little’s MCAR test *x*^2^ = 71.86, DF = 63, *p* = .21) and were therefore excluded from the current study. Descriptive statistics were provided for all 1,793 participants included in the study and separately for each latent class. Chi-square tests and one-way analyses of variance were conducted to identify significant differences between the latent classes and covariates. Correlation coefficients were also calculated to assess the strength of the relationship between the covariates and self-control. Analysis of covariance was then conducted to compare the adjusted mean self-control score between the latent classes independent of covariates. The Bonferroni correction was applied to post-hoc comparisons. Two-tailed significance tests (*p* < .05) were used, and the assumptions underlying all statistical analyses were met. Analyses were conducted using SPSS v.26 (IBM, 2019).

## Results

### Latent Classes of Cyber-Deviance

**Figure 1.**
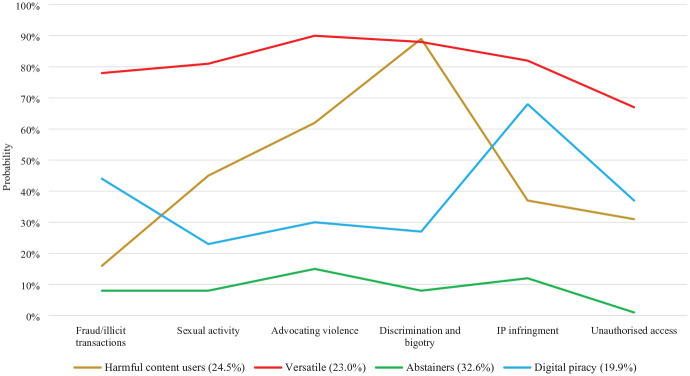
Four model solution for latent profiles of cyber-deviance (*n* = 1,793).

Test statistics from the latent class analysis indicated that the four-class solution was the optimal model (see Table 1), revealing four clearly distinguishable classes (see Figure 1). A quarter of participants were assigned to the “*harmful content users”* (*n* = 439, 24.5%) group, which was characterized by a high probability of engagement in discrimination and bigotry, as well as advocating violence, but a moderate to low probability of engagement in other forms of cyber-deviance. Around a quarter of participants demonstrated a high probability of engaging in all six forms of cyber-deviance and were labelled as the “*versatile”* (*n* = 413, 23.0%) group. The “*abstainers”* (*n* = 585, 32.6%) class was the largest group, and encompassed participants who had the lowest probability of engagement in any type of cyber-deviance. Finally, the smallest group, designated “*digital piracy”* (*n* = 356, 19.9%), had a moderately high probability of engaging in intellectual property infringement, but a moderate to low probability of committing all other types of cyber-deviance. Table 2 details the item probabilities (rho estimates) for each type of cyber-deviance across the four latent classes.

**Table 1.** Latent Class Analysis test statistics.

NO. classes	Likelihood ratio *G*^2^	*df*	AIC	BIC
2	214.03	50	240.03	311.42
3	111.89	43	151.89	261.73
4	32.44	36	86.44	234.71
5	21.57	29	89.57	276.29

**Table 2.** Item Response Probabilities (Rho Estimates) for the Four-Class Solution.

Scale	Class 1	Class 2	Class 3	Class 4
	Abstainers (*n* = 585; 32.6%)	Harmful content users (*n* = 439, 24.5%)	Digital piracy (*n* = 356; 19.9%)	Versatile (*n* = 413, 23.0%)
Fraud/illicit transactions	.08	.16	.44	.78
Sexual activity	.08	.45	.23	.81
Advocating violence	.15	.62	.30	.90
Discrimination and bigotry	.08	.89	.27	.88
IP infringement	.12	.37	.68	.82
Unauthorized access	.01	.31	.37	.67

### Descriptive Statistics and Univariate Analyses

Table 3 presents the descriptive statistics for the total sample and each latent class. Around half of all students were male (49.3%), most identified as Caucasian (70.8%), and one-in-five experienced socioeconomic disadvantage (21.4%). Self-control (*range* = 1–4), unsupervised internet use (*range* = 0–4), and digital device engagement (*range* = 0–5) scores were normally distributed, with the average in the middle of the score range. In the last 12 months, just under half (45.5%) of all students had engaged in some form of antisocial behavior offline, whereas 80.7% (*n* = 1447) had engaged in at least one type of cyber-deviance. Just under half of all students had engaged in both offline antisocial behavior and cyber-deviance (*n* = 764, 42.6%).

**Table 3.** Descriptive Statistics (*n* [%]/*m* [*SD*]).

Variables	Total (*N* = 1,793)	Cyber-deviance profiles			
		Abstainers (*n* = 585)	Harmful content users (*n* = 439)	Digital piracy (*n* = 356)	Versatile (*n* = 413)
Self-control	2.55 (0.59)	2.34 (0.60)	2.57 (0.52)	2.59 (0.59)	2.78 (0.53)
Covariates					
Male	884 (49.3%)	269 (46.6%)	165 (37.8%)	216 (61.2%)	234 (57.9%)
Caucasian	1,270 (70.8%)	413 (70.6%)	318 (72.4%)	249 (69.9%)	290 (70.2%)
Socioeconomic disadvantage	383 (21.4%)	113 (19.3%)	98 (22.3%)	77 (21.6%)	95 (23.0%)
Offline antisocial behavior	814 (45.4%)	108 (18.6%)	207 (47.3%)	169 (47.7%)	330 (80.3%)
Unsupervised internet use	2.21 (0.94)	1.96 (1.02)	2.30 (0.86)	2.24 (0.90)	2.45 (0.86)
Digital device engagement	2.84 (1.10)	2.40 (1.13)	3.09 (1.04)	2.89 (0.99)	3.33 (0.97)
Cyber-deviance scales					
Fraud/illicit transactions	615 (34.3%)	39 (6.7%)	17 (3.9%)	200 (56.2%)	359 (86.9%)
Sexual activity	660 (36.8%)	49 (8.4%)	184 (41.9%)	80 (22.5%)	347 (84.0%)
Advocating violence	829 (46.2%)	81 (13.8%)	255 (58.1%)	112 (31.5%)	381 (92.3%)
Discrimination and bigotry	881 (49.1%)	0	437 (99.5%)	75 (21.1%)	369 (89.3%)
IP infringement	840 (46.8%)	85 (14.5%)	160 (36.4%)	254 (71.3%)	341 (82.6%)
Unauthorized access	563 (31.4%)	0	111 (25.3%)	170 (47.8%)	282 (68.3%)

There were significant differences between the cyber-deviance groups regarding the proportion of students who were male (χ^2^ = 56.22, Ф = .18, *p* < .001) and had engaged in antisocial behavior offline in the last 12 months (χ^2^ = 372.13, Ф = .46, *p* < .001). The average time unsupervised on the internet (*F* [3,1778] = 24.08, *p* < .001) and frequency of digital device engagement (*F* [3,1784] = 72.33, *p* < .001) also significantly differed between the cyber-deviance groups. There were no significant between group differences in socioeconomic disadvantage (χ^2^ = 2.38, Ф = .04, *p* *=* .49). Pearson and Point-biserial correlation coefficients indicate that self-control had a very weak correlation with race (*r*_pb_ = .07, *p* = .003), a weak correlation with gender (*r*_pb_ = .12, *p* < .001) and unsupervised internet use (*r* = .12, *p* < .001), a weak approaching moderate correlation with digital device engagement (*r* = .22, *p* < .001), and a moderate approaching strong correlation with offline antisocial behavior (*r*_pb_ = .31, *p* < .001) (Funder & Ozer, 2019). Socioeconomic disadvantage was not significantly correlated with self-control (*r*_pb_ = −.02, *p* = .36).

### Low Self-Control

One-way analysis of variance indicated that there were significant differences in observed self-control scores between the cyber-deviance groups (*F* [3,1749] = 49.50, *p* < .001, η^2^ = .08). Bonferroni corrected post-hoc comparisons demonstrate that the *abstainer* group had significantly higher self-control on average (*m* = 2.34, *se* = .02) than the *harmful content users* (*m* = 2.57, *se* = .03, *p* < .001, *d* = .41), *digital piracy* (*m* = 2.59, *se* = .03, *p* < .001, *d* = .42), and *versatile* (*m* = 2.78, *se* = .03, *p* < .001, *d* = .78) group. The *versatile* group also had significantly lower self-control scores on average than the *harmful content users* (*p* < .001, *d* = .40) and *digital piracy* (*p* < .001, *d* = .34) groups. There were no significant differences between the *harmful content users* and *digital piracy* groups (*d* = .04).

Interaction effects were calculated to assess the independence between the latent classes and covariate factors. Non-significant interactions were found for gender (*F* [3,1723] = 0.82, *p* = .48), race (*F* [3,1745] = 0.45, *p* = .72), socioeconomic disadvantage (*F* [3,1745] = 0.99, *p* = .40), digital device engagement (*F* [3,1740] = 0.40, *p* = .76), unsupervised internet use (*F* [3,1734] = 0.27, *p* = .85), and offline antisocial behavior (*F* [3,1742] = 0.36, *p* = .78), indicating that between-group differences in self-control were not a function of the covariates.

**Figure 2.**
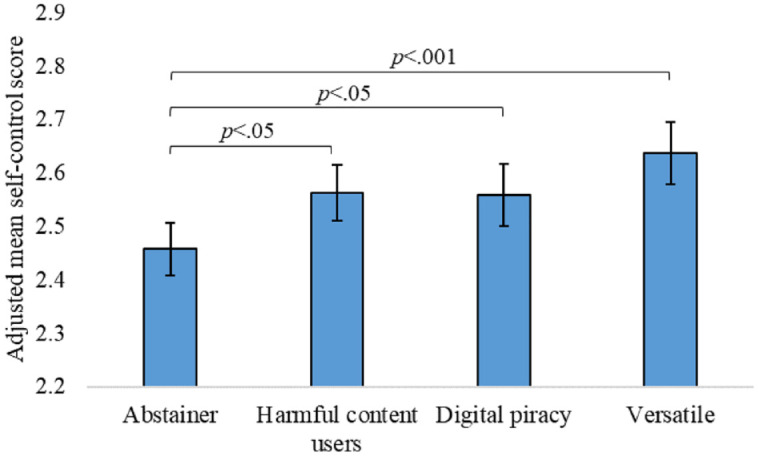
Adjusted mean self-control score by latent class.

The results of the analysis of covariance are presented in Table 4 and Figure 2 and show that there were significant differences in self-control scores across the latent classes after adjusting for covariates (*F* [3,1704] = 5.72, *p* < .001). Bonferroni corrected post-hoc comparisons indicate that the adjusted mean self-control score was significantly higher for the *abstainer* group (adjusted mean = 2.46, std. error = .02) than for the *harmful content users* (adjusted mean = 2.56, std. error = .03, *p* = .03, *d* = .18), *digital piracy* (adjusted mean = 2.60, std. error = .03, *p* = .05, *d* = .18), and *versatile* (adjusted mean = 2.64, std. error = .03, *p* *<* .001, *d* = .31) group. The *versatile* class did not significantly differ from the *harmful content users* (*d* = .13) or *digital piracy* (*d* = .14) class.

**Table 4.** Analysis of Covariance for Risk-Taking Behavior Score by Latent Class With Covariates.

	*SS*	*Df*	*MS*	*F*	*p*	η^2^
Latent class	5.72	3	1.91	6.43	<.001	.011
Gender	10.83	1	10.83	37.15	<.001	.021
Race	0.84	1	0.84	2.87	.09	.002
Socioeconomic disadvantage	0.57	1	0.57	1.95	.16	.001
Digital device engagement	14.06	1	14.06	48.25	<.001	.028
Unsupervised internet use	1.95	1	1.95	6.70	.01	.004
Offline antisocial behavior	17.67	1	17.67	60.62	<.001	.034
Error	496.69	1,704	0.29			

*Note. R*^2^ = .162 (adjusted *R*^2^ = .157).

## Discussion

To date, no study has examined the association between low self-control and distinct patterns of cyber-deviance independent of offline antisocial behavior, opportunities for online deviance, and demographic characteristics. The current study addressed this research gap by examining if self-control was differentially associated with distinct latent classes of adolescent cyber-deviance net of potential confounders among a cross-sectional sample of 1,793 South Australian adolescents. Four latent classes were identified, each with varying probabilities of involvement in six types of cyber-deviance. The *abstainer* (32.6%) class, defined as having a low probability of engagement in any cyber-deviance, had the highest average self-control. Lower self-control was evidence in the *harmful content users* (24.5%) and *digital piracy* (19.9%) groups, which were characterized as having a high probability of engagement in advocating violence and intellectual property infringement, respectively. Finally, the *versatile* (23.0%) class had a high probability of engagement in all types of cyber-deviance and had the lowest average self-control.

Results indicate that those who abstained from cyber-deviance had significantly higher self-control than other classes of cyber-deviance after adjusting for offline antisocial behavior, frequency of digital device engagement, time spent unsupervised online, gender, race, and socioeconomic disadvantage. This is consistent with other studies showing independent links between low self-control and engagement in specific or any type of cyber-deviance, relative to those who refrain from the behavior (Bae, 2017, Holt et al., 2012; Li et al., 2016; Marcum, Higgins, & Ricketts, 2014; Marcum, Higgins, Ricketts, & Wolfe, 2014). Unique to this study, however, is the finding that self-control did not significantly differ between other patterns of engagement in cyber-deviance after accounting for covariates. Although the *versatile* class had noticeably lower average self-control scores than the *harmful content users* and *digital piracy* groups, this difference was not significant after correcting for multiple comparisons. Collectively, these findings suggest that self-control appears to distinguish between those who do and do not engage in cyber-deviance but may not distinguish between distinct patterns of cyber-deviance net of confounding factors.

A key gap that our paper addresses is that, while prior research has used LCA to discern clusters distinguishing the frequency of a *single* type of cyber-deviance, no other study has used this analysis to identify distinct patterns of the probability of engagement across *multiple* types of cyber-deviance. Hence it would be inappropriate to extrapolate our findings to other studies using LCA, such as those by Cho and Glassner (2021) or Tian et al. (2023), for example, because they identified latent classes characterized by the longitudinal frequency of cyber bullying specifically, and not on the cross-sectional probability of engagement across multiple types of cyber-deviance. In other words, the shapes and characteristics of the latent classes cannot be compared because of differences in the unit of measurement (frequency of engagement vs. probability of engagement), methodology (longitudinal vs. cross sectional), research aims, and types of cyber-deviance. As such, more research identifying young people’s patterns of engagement across multiple types of cyber-deviance is needed to determine if our findings are replicable to other adolescent samples.

Consistent with other studies, we found a substantial overlap between engagement in cyber-deviance and antisocial behavior offline, the latter being the strongest correlate of cyber-deviance (e.g., Kim et al., 2017; Seigfried-Spellar et al., 2017). However, our multivariate models indicate that it did not completely account for the association between low self-control and cyber deviance. The lack of a significant interaction effect also suggests that the association between low self-control and cyber-deviance did not differ for those who did and did not engage in antisocial behavior offline. Therefore, low self-control may be an indicator of cyber-deviance regardless of engagement in antisocial behavior offline. This is consistent with Gottfredson and Hirschi’s (1990) argument that low self-control underlies all forms of antisocial behavior, although the specific type of antisocial behavior one engages in is also influenced by situational circumstances. Therefore, one possibility is that those with fewer opportunities for antisocial behavior offline may be more inclined to engage in cyber-deviance, in the context of an abundance of opportunities to do so, although more research examining this hypothesis is required.

Our findings also indicate that the association between self-control and the latent classes of cyber-deviance was not influenced by between-group differences in unsupervised internet use and digital device habits, as indicated by non-significant interaction effects. This potentially suggests that the link between low self-control and engagement in specific patterns of cyber-deviance may not be conditional on opportunities for online misconduct. This contrasts with Gottfredson and Hirschi’s (1990) argument that variations in opportunities for misconduct affect the likelihood that low self-control will result in antisocial behavior. An alternative explanation may be that, because there are more opportunities for online cyber-deviance than offline antisocial behavior, the opportunity for online misconduct may be less instrumental in determining the specific type of cyber-deviance one engages in. Instead, certain individual characteristics, such as psychopathology or digital device skills, may better moderate engagement in specific types of online misconduct (Payne et al., 2019; Seigfried-Spellar et al., 2015).

Given our findings, and those of the broader literature, low self-control appears to be ubiquitous across all types of cyber-deviance, and therefore may be a key target for cyber-deviance intervention. This could include school-based childhood and early adolescent self-control programs, which appear to elicit modest improvements in self-control and reductions in delinquency (Piquero et al., 2016). Interventions specifically focusing on adolescent psychosocial development, such as socio-emotional learning (SEL) programs, may also be useful given they are associated with improved behavioral regulation, impulse control, and fewer conduct problems (Coelho & Sousa 2017; Durlak et al., 2011; Greenberg et al., 2003). Although only a small number of cyber-deviance interventions have included SEL components in their design, those that have done so have shown a reduction in adolescent cyber-bullying and aggression (Espelage et al., 2015; Garaigordobil & Martínez-Valderrey, 2018).

Because the findings of this current study are correlational and not causal, it is not possible to attest to the predictive utility of our results for the prospective identification of at-risk youth. However, given that there appears to be relative stability in self-control from childhood to adolescence (Coyne & Wright, 2014), it is possible that low self-control may be a potential risk factor for early adolescent engagement in any cyber-deviance, independent of other factors. Strategies to strengthen children’s behavioral regulation and impulse control may therefore be useful for decreasing their risk of engagement in cyber-deviance. Although Gottfredson and Hirschi (1990) contend that such efforts ought to be conducted by parents during early childhood, evidence suggests that clinical and school-based self-control programs are also effective at improving self-control and decreasing delinquency outcomes (Piquero et al., 2016). Despite this conclusion, more research is needed to examine the causal contribution of low self-control on distinct patterns of cyber-deviance, and the influence of offline antisocial behavior and opportunities for online misconduct on this potential pathway.

To our knowledge, this is the first study to identify distinct patterns of engagement in cyber-deviance, thereby addressing an important gap in the literature. This study also has the benefit of using a large sample of Australian adolescents, and an array of detailed and reliable measures of online and offline behavior. However, our findings must be interpreted within the context of the study limitations. Foremost, the DYS only captured around one-quarter of Grade 8 students enrolled in Government schools within the catchment area. Our findings may therefore not be generalizable to the adolescent population, particularly those who attend non-government schools (e.g., private schools) or reside in more rural areas. Second, the latent classes identified reflect approximations of the *likelihood* of engagement in patterns of cyber-deviance and are not to be interpreted as literal entities. The shape and number of latent classes may differ across samples, and therefore more research examining the generalizability of these patterns are needed. Third, this was a cross-sectional study, and therefore no causal inferences can be made. Finally, it is acknowledged that this study did not examine the effect of other factors strongly correlated with cyber-deviance, such as parenting quality, social bonds, deviant peer association, attitudes toward cyber-deviance, and academic performance, as these variables were not measured in the DYS.

The internet, and digital technologies more broadly, provide an additional platform for the commission of crime and antisocial behavior that is absent of many conventional deterrents, thereby providing a reduced risk for repercussion. More empirical attention is needed regarding young people’s engagement in patterns of cyber-deviance, as well as the associated developmental precursors. Greater awareness should also be placed on the potential utility of self-control in understanding the development and potential overlap of engagement in antisocial behavior offline and cyber-deviance. Indeed, more research should be devoted to identifying the risk factors and causal mechanisms that overlap and distinguish online and offline forms of deviance to develop better informed intervention programs.
